# Creating a Person- and Family-Centered Program of Research: Lessons Learned From Over 30 Years of Applied Research

**DOI:** 10.1093/geroni/igab046.006

**Published:** 2021-12-17

**Authors:** Carol Whitlatch, Silvia Orsulic-Jeras

**Affiliations:** 1 Benjamin Rose Institute on Aging, Shaker Heights, Ohio, United States; 2 Benjamin Rose Institute on Aging, Cleveland, Ohio, United States

## Abstract

Approximately 6 million Americans are living with Alzheimer’s Disease or related dementia. Due to these alarming statistics, there is an increased need for families to seek out services and supports to not only cope with these devastating diagnoses, but to plan effectively for their future care needs. A plethora of research has shown that both the family care partner and person living with dementia are at-risk for negative outcomes such as depression, anxiety, social isolation, and worsening physical and mental health. Moreover, further and encouraging research supports the development and implementation of empowerment-based, person- and family-centered interventions. When utilized effectively these interventions improve quality of care and well-being in persons living with dementia and their care partners. The purpose of this paper is to provide guidance for researchers interested in making their work more person- and family-centered. Strategies discussed are based on over 30 years of applied research and include: 1) placing individuals at the center of their own care, 2) including persons with dementia as co-investigators, 3) convening diverse professionals and individuals in advisory councils from the start, and 4) conducting focus groups to obtain participant and stakeholder feedback. Demonstrations of select person-and-family-centered, evidence-programs will be included and supplemented with case examples to illustrate person-centered principles in practice.

